# Per-chloride of Iron in Erysipelas

**Published:** 1862-01

**Authors:** 


### Selections.

### PER-CHLORIDE OF IRON IN ERYSIPELAS.

Reprinted from *American Journal of Medical Sciences.*

The No. of the *Edinb. Med. Jour.* for July last, contains some interesting remarks by Dr. Wm. Pirrie, Jr., on Erysipelas and its treatment by per-chloride of iron. "As the exact nature of the poison of erysipelas," he observes, "is unknown, we are not in possession of any remedy having the virtues of an antidote. Neither can any single plan of treatment, yet devised, be recommended as equally applicable to all cases, inasmuch as the features of many greatly differ, owing perhaps to the particular diathesis of the subject, or to his state of general health, and the circumstances he was placed in at the time of seizure, or to the peculiar type of the prevailing epidemic. That the last-named circumstance requires to be carefully weighed in every instance, will at once be readily granted; for one visit to the majority of the cases of erysipelas of the head and face, as now met with, will at once convince us that they are altogether unsuited for the blood-letting and other powerful antiphlogistic remedies which seem to have been so beneficial, according to the records of bygone observers.

On the other hand, that the excessive stimulation so strongly advocated by some, for every case of the disease in question, is not an indispensable remedial agency for successful treatment in most instances, may, I humbly think, be reasonably inferred from the multitudes of recoveries which have taken place under a more moderate use of stimulants.

The conviction that erysipelas generally tends to a spontaneous recovery is by no means incompatible with holding the belief, that much may be done by suitable treatment to check the intensity of the disease, to alleviate or remove many of its most distressing symptoms, to maintain the vital powers, to moderate inflammatory action, and to assist the natural efforts at cure, and thus more surely conduct the disease to a favorable termination. Examples of the disease often occur, which from the first present a very unfavorable aspect, yet issue in recovery. Such recovery we may justly ascribe to the line of treatment pursued. It is, therefore, the duty of every one to place on record any plan of treatment which he thinks has been attended with success, and allow it to get a fair trial in the hands of others; to state fairly the effects which appeared to him to follow the employment of any particular remedy, in what respects it modified the intensity of the disease, and what symptoms it alleviated or controlled; without trying to deny that much is done for recovery in this disorder by the spontaneous operations of nature. It has always appeared to me, that in a careful study of this, as well as other diseases, we discover certain landmarks or beacons, as it were, to guide us to the adoption of a scientific and rational method of treatment. We find, for example, that the disease cannot be cut short by any known agency, that it must run a definite course, that it early produces a most depressing effect on the system, that there are symptoms more particularly distressing than others to the sufferer, and that in due time nature employs a machinery of her own for the removal of the noxious matter from the system.

The great indications of treatment then, suggested by a consideration of these points, are—to uphold the powers of the system, to ameliorate or control the most distressing symptoms, to obviate the tendency to death, and to assist, if possible, the natural efforts at cure.

The first indication is to be fulfilled by removing as speedily as possible any existing internal sources of irritation, by correcting any hepatic or alvine disorder, by rigidly enforcing thorough ventilation, with due regard to the maintenance of an equable temperature and guarding against exposure to currents, and by giving some medicine or remedies capable of imparting tone to the system and of upholding the vital powers. A marked feature of the disease, as already mentioned, is early depression of the various parts of the organism—the energy of the nervous system becoming rapidly exhausted—the vigor of the muscular system being speedily impaired—the tone of the vascular system lowered—the capillaries relaxed, and serous effusions quickly ensuing. I was induced, by the high terms in which many have written in favor of the muriated tincture of iron, recommended by Dr. Hamilton Bell, for the treatment of erysipelas, to try what effect the per-chloride of iron might have on the disease. Having given it a fair trial in five successive cases, I will simply state the effects which I observed to follow, in the hope that others may give it a trial, and thus an opportunity be obtained of comparing the effects produced in different cases, and the means be acquired of arriving at some definite understanding as to its value in the generality of cases. In all the cases in which I used it, a decidedly beneficial effect seemed to be produced. The febrile condition seemed in all to be relieved, the frequency of the pulse reduced, the powers of the system generally upheld, and the stomach and bowels in no way irritated. Two circumstances I would particularly notice with regard to the perchloride in the cases in which I tried it. One, which I carefully noticed was, that its use seemed in no way to be contraindicated by headache and sensorial disturbance; instead of increasing, it seemed to diminish these unpromising symptoms. The other circumstance to which I would particularly direct attention is, that in the above cases the serous effusion was, throughout the entire course of the disease, less copious, and also disappeared much more quickly, than in the generality of equally grave examples which I had previously seen.

Two persons, who had a very severe attack, stated to me spontaneously, without my directing their attention in any way to the subject, that they very speedily felt a most decided general benefit from its use; and the others, on inquiry, said they 'felt it keep down the fever' and speedily diminish the 'tightness' of their faces.

The taste of the per-chloride is to some very unpleasant, but it may be quickly removed by thoroughly washing out the mouth with water immediately after taking the solution. Special attention ought to be paid during its employment, to act on the bowels from time to time by some very mild laxative. What its specific mode of action is, I do not pretend exactly to know. Perhaps it may act beneficially, in virtue of powerful tonic properties, or its therapeutic value may reside in some peculiar influence it exerts on the blood, in consequence of its powerful disinfectant properties, or in its imparting tone to the capillary system, and counteracting the general relaxation of the capillary vessels. In the cases in which I used the medicine, I ordered it to be taken in quantities varying from fifteen to twenty drops every two and a half or three hours, according to the peculiarities of each case. This was persevered with till convalescence was fairly established, after which the iron was greatly reduced, and drachm and a half, or two drachm doses of liquor ammoniæ acetatis ordered to be taken three or four times a day at proper intervals. The liquor ammoniæ acetatis acts very beneficially in the convalescent stage, by being a very gentle stimulant to the nervous and vascular systems, a mild diaphoretic, and an unirritating diuretic; and thus, by gently stimulating the functions of the skin and kidneys, it fulfils the last-named indication of treatment. In the course of any attack of erysipelas, just as in all fevers, the lowering effect of the disease may become so great, and the tendency to death, from failure of the vital powers, so strongly marked, as imperatively to call for the use of powerful stimulants. The employment of these agents, in this and in all diseases, to be beneficial, requires the greatest prudence and judgment in their selection and apportionment, and the most careful watching of the effects they produce. The exact form of stimulant, whether wine, brandy, or other spirit, must be determined by the particular situation of each individual case, and the previous habits of the patient.

The delirium and coma, which so often accompany erysipelas, are, according to general consent, the result of diminution or arrest of the cerebral energy, caused by mal-nutrition of the brain substance, and to be treated most successfully by the cautious use of stimulants. Cases, however, may occur in which some of the symptoms of genuine phrenitis appear, and then it might be judicious to employ some form of local depletion,

The bronchitis attacks are to be treated by mustard cataplasms, blisters, or turpentine stupes to the chest; but the two latter I have once or twice seen irritate the renal organs very much in this disease. Should the attack supervene when the system is very low, stimulants must be given; but if during the period of convalescence, some simple stimulating expectorant, in addition to the counter-irritation, generally suffices for cure. If symptoms of œdematous effusion into the submucous tissue of the glottis and epiglottis arise, the speedy performance of tracheotomy affords almost the only chance of saving life. Of all local applications, perhaps the most serviceable and least objectionable, under all states of the skin, are flannels wrung out of hot water. This application, no doubt, is open to the objection of requiring almost constant attention, and of occasioning considerable trouble for its proper employment; but its advantages in many respects over unguents, dry powders, or other appliances, more than compensate for these slight drawbacks. When proper precautions are taken to maintain a uniform heat, by frequent renewal of the flannels, the application possesses the great advantages of being cleanly, generally agreeable to the feelings of the sufferer, unirritating to the skin, calculated to prevent any repression of the external inflammation, and not forming, as unguents and powders do, with any exudation which may have taken place, the scabs and crusts which become sources of great local irritation, and general discomfort to the invalid.

